# Evaluating a biochemical TB medication adherence test: Insights from image-based reporting through a patient companion app

**DOI:** 10.1371/journal.pgph.0007182

**Published:** 2026-10-05

**Authors:** Kiara Lee, Enrique Chen Liang, Alejandra Tolentino, AuDuyen Trinh, Daniel Leon, Kyle Goodwin, Jennifer Sprecher, Fernando Rubinstein, Barry Lutz, Sarah Iribarren

**Affiliations:** 1 Engineering Department, Bioengineering Section, Pontificia Universidad Católica del Perú, Lima, Peru; 2 University of Washington, Department of Biobehavioral Nursing and Health Informatics, Seattle, Washington, United States of America; 3 University of Washington, Department of Bioengineering, Seattle, Washington, United States of America; 4 Instituto de Efectividad Clinica y Sanitaria (IECS), Buenos Aires, Argentina; PLOS: Public Library of Science, UNITED STATES OF AMERICA

## Abstract

Tuberculosis (TB) treatment requires strict adherence over the long course of treatment. To support adherence, we developed the TB Treatment Support Tools (TB-TST), which combines a urine-based lateral flow assay (LFA) with a mobile application for patient education, support, and communication with treatment supporters. In a pragmatic randomized controlled trial participants uploaded images of the LFA. A blue-purple color change indicates detection of an isoniazid metabolite consistent with recent medication ingestion. This study assessed real-world interpretability and usability of the TB-TST LFA by analyzing the quality and characteristics of user-submitted images. We conducted structured qualitative coding (visual content analysis) of 1,655 submitted test images to identify recurring issues affecting interpretation. Eligible participants were aged 16 or older, newly diagnosed with TB, and had access to a mobile phone. An iteratively developed codebook identified three primary issue categories: 1) color variations in the test or control strip, 2) photo quality issues, and 3) test cartridge/test-strip artifacts. Most (72%) images were consistent with a clear positive (blue-purple) color change or clear negative. Variations that were not clear positives included no color change (white), yellow/orange, and green/teal hues. Blurriness was the most common image quality issue. The most common test artifact was visual evidence consistent with dye bleed/possible cross-strip contamination between the control and the test strips. Despite challenges in image submission and test appearance, relatively few images were difficult to interpret, supporting the feasibility of user-submitted LFA images for monitoring recent isoniazid ingestion. Opportunities to improve performance and usability include enhanced user guidance for image capture, refinement to cartridge design to reduce artifacts, clearer interpretation protocols for atypical color outcomes, and automated image-based reading. Integrating qualitative image analysis with engineering design principles can inform user-centered improvements to home-based LFAs connected to digital adherence technologies.

## 1. Background

Tuberculosis (TB) remains the world’s leading cause of death from a single infectious agent, despite being preventable and curable [1]. Effective treatment for drug-susceptible TB requires strict adherence to a six- to nine-month multi-drug antituberculosis treatment regimen. Historically, the World Health Organization (WHO) recommended directly observed therapy (DOT), in which healthcare providers supervise medication intake [2]. While DOT has improved adherence in some settings, it is logistically burdensome for both patients and healthcare systems, limiting scalability and accessibility; current WHO guidelines now emphasize patient-centered care approaches [3]. Maintaining adherence is critical to improve cure rates, reduce transmission, prevent TB-related mortality, and minimize the emergence of drug-resistant TB strains [4]. However, existing adherence monitoring strategies often lack flexibility, accuracy, and patient-centered design [3], underscoring the need for remote, scalable solutions [5].

Digital Adherence Technologies (DATs) have shown promise in enhancing TB treatment adherence, with randomized controlled trials demonstrating benefits from video monitoring, phone-based communication, SMS reminders, and ingestible sensors [4]. Among these, video monitoring and ingestible sensors have shown the highest potential, however video monitoring requires reliable internet access, and ingestible sensors remain cost-prohibitive in many settings [4]. With patients and TB experts, we co-developed the TB Treatment Support Tools (TB-TST) intervention, which combines an objective biochemical adherence test with a mobile application for patient education, support, and communication with treatment supporters [6,7].

A key component of the TB-TST intervention is the biochemical adherence test, a colorimetric lateral flow assay (LFA) based on the Arkansas reaction [8–10], which detects isoniazid metabolites in urine and produces a visible color change within 24 hours of medication ingestion. Isoniazid is a principal first-line TB drug and is often combined with other antibiotics into a single tablet to simplify treatment and improve adherence. Studies with tests operated by healthcare providers have shown excellent correspondence with a commercial test [9]. For our study, we modified the test to make it easier for patients to operate at home, and we developed image capture for patients to upload a photo of their test result via a mobile application, allowing remote adherence monitoring and enabling treatment supporters to follow-up with individuals who may be struggling to maintain treatment adherence. In a pragmatic randomized clinical trial across four large public hospitals in Argentina, the TB-TST intervention significantly improved treatment success (81.6% vs 74.4%) and reduced loss to follow-up (17.2% vs 24.4%) compared to standard of care (both p = 0.04) [11–13]. These findings demonstrated the feasibility of adapting a laboratory-based, clinician-operated assay into a self-administered home test for remote treatment monitoring.

LFAs are widely used due to their versatility, affordability, portability, and ease-of-use [14]. While traditionally applied for condition detection (e.g., pregnancy, COVID-19, etc.), their potential for medication adherence monitoring remains underexplored [9]. Some LFAs have been developed for antiretroviral therapy adherence in HIV and validated in randomized controlled trials [15]. Additionally, smartphone-based image analysis has been investigated for fluorescence detection, result interpretation, and colorimetric analysis [16–19].

The TB-TST offers a practical alternative to frequent in-person DOT by allowing patients to submit data remotely in under 20 minutes. Similar smartphone-based, image-based applications have been successfully used to submit test results or images for anemia [20,21] and diabetic foot ulcer monitoring [22], yet few studies have systematically analyzed variations in user-submitted images.

To optimize this technology, we conducted a qualitative evaluation (visual content analysis) of user-submitted images from the pragmatic clinical trial [12]. The objectives of this study were to identify color interpretability of the user-submitted images and assess for user-related errors and test-related defects of the modified home-based LFA. This manuscript presents our analysis, highlighting real-world challenges and proposing design and process modifications to improve usability and reliability. Our findings contribute to the refinement of smartphone-compatible LFAs for medication adherence monitoring, demonstrating their potential for TB treatment support.

## 2. Methods

### 2.1. Ethics statement

The study protocol approved by the Institutional Review Board of the province of Buenos Aires, Argentina (2919-1891-2019-0) and the University of Washington, USA (STUDY00007533), with additional approvals obtained from each participating hospital. All participants provided written informed consent prior to participation

### 2.2. Study design

This study was a qualitative secondary analysis of data from a pragmatic randomized clinical trial evaluating the effectiveness of the TB-TST intervention. The trial was registered at ClinicalTrials.gov (NCT04221789) and International Registered Report Identifier (DERR1-10.2196/28094). We analyzed user-submitted test images collected between November 21, 2020, through July 31, 2023, to identify issues related to test usage and image quality and identify opportunities for improvement. The analysis team did not have access to information that could identify individual participants after data collection.

#### TB medication adherence test overview.

The colorimetric TB Medication Adherence Test consists of a test strip, a control strip, a cartridge, and a cover sticker (Fig 1). The test detects isoniazid metabolites in urine via the Arkansas reaction [8], producing a color change from white to blue-purple. Two key indicators ensure accurate interpretation: a control strip confirms sufficient urine uptake for reaction completion, and the test strip indicates the presence of isoniazid metabolites. Participants could complete the test by either voiding directly onto the designated void window or dipping it into a urine sample. While color change may appear within minutes, users were instructed to lay the test flat for up to 20 minutes to ensure full reaction completion.

**Fig 1:**
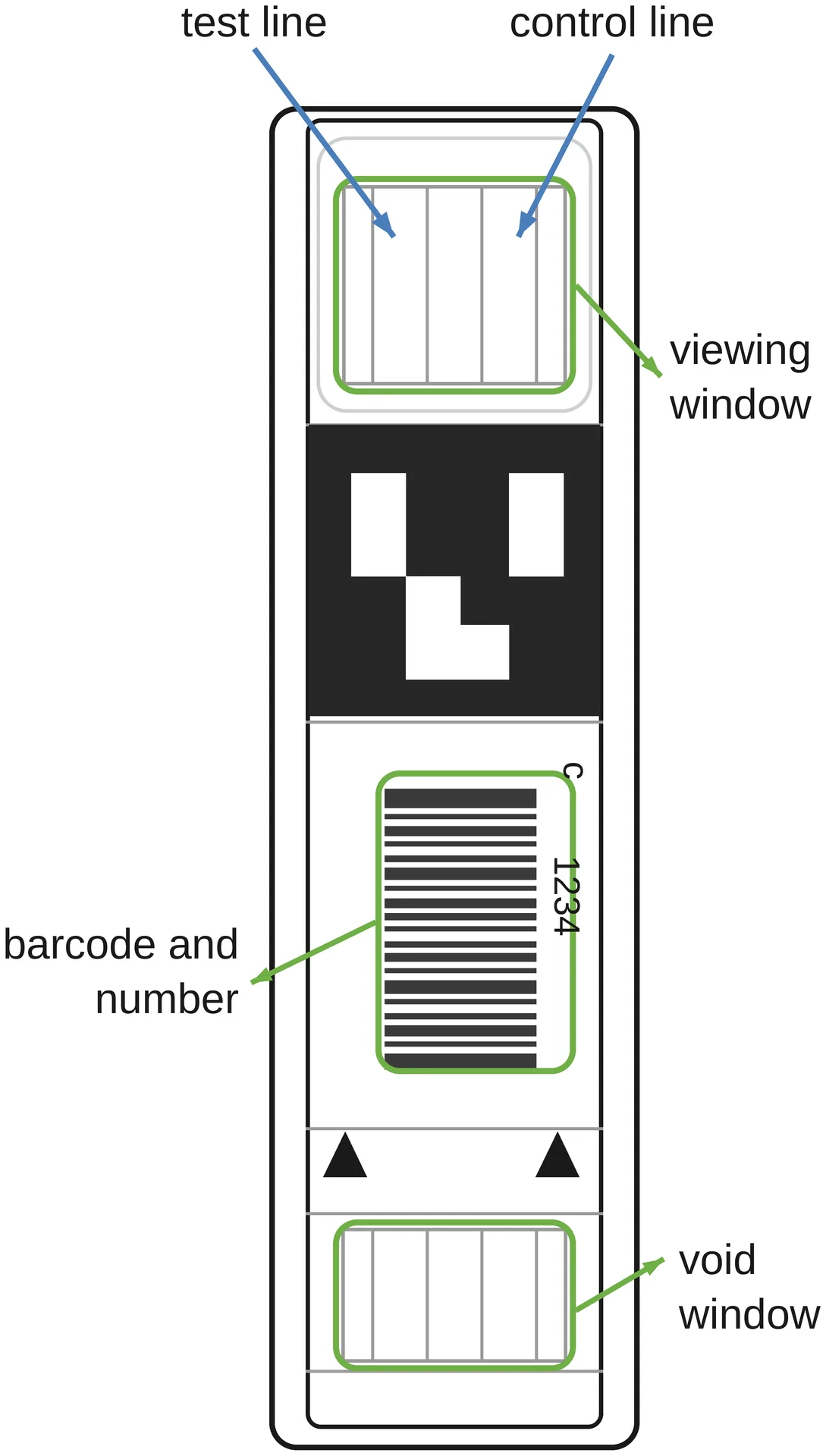
TB medication adherence test design.

The test was designed with built-in features to facilitate digital processing. The cover sticker includes an ArUco marker for image orientation and automated test component recognition. The barcode allows unique test identification and production tracking.

### 2.3. Data analysis

A multi-step iterative pragmatic analytic approach was used to develop the codebook, which was subsequently systematically applied to all images. The categories of images were developed by the analytic team (KL, EL, AT) and iterated on with other research team members (SI) during regular team meetings. An initial subset of images (70 images) was reviewed independently by four bioengineering researchers, who further defined the finalized issue categories: (1) color variations in the test or control strip, (2) photo quality issues, including blurriness, incomplete visibility, off-center framing or improper distance, and (3) test cartridge issues, such as sticker damage or misalignment of components. Multiple issues could be coded for a single image. Initial iterations of the codebook attempted to define each color as a separate category and were re-worked to be focused on result interpretation. Through discussion and meetings to reach consensus, a refined codebook was finalized (S1 File). Two researchers (EL, AT) independently coded the full dataset using Microsoft Excel (Microsoft Corporation, Inc., Washington, USA). They each split the images evenly to each independently code a subset and double coded every ~20^th^ image to ensure consistency and reliability. Descriptive statistics were used to determine the frequency of each subset of issues identified. Findings were synthesized to: (1) identify test design flaws, (2) explore potential underlying causes, and (3) propose design modifications to address identified issues.

## 3. Results

### 3.1. Study setting and participants

Enrollment occurred on a rolling basis at four large public hospitals in Argentina with high TB caseloads. Participants were individuals randomized to the intervention group. Eligibility criteria included being aged 16 and older, newly diagnosed with drug-susceptible TB, and having access to a smartphone. Participants in the intervention group were assisted with downloading the app and provided verbal and written instructions, including a demonstration of key app features and how to complete the urine metabolite test and submit a photo. They were instructed to log daily medication intake, report potential side effects, and submit weekly urine test results when prompted via a smartphone push notification on a randomly assigned day. Treatment supporters reviewed the test submissions and followed up as needed.

Participant characteristics are found in Table 1.

**Table 1:** Participant sociodemographic characteristics.

Characteristic	N = 252	
Sex, n (%)	Female	134 (53%)
	Male	117 (47%)
Age, mean, (sd)	Mean Age	33.2 (13.66)
Steady Employment, n (%)	Yes	131 (52%)
	No	121 (48%)
Income Level, n (%)	At or below the Poverty line	159 (63%)
	Above the Poverty line	71 (28%)
	No Answer	22 (9%)
Married/partner, n (%)	Yes	139 (55%)
	No	113 (45%)
Argentine National, n (%)	Yes	193 (77%)
	No	59 (23%)

### 3.2. Overview of images analyzed

A total of 2,447 images were submitted during the study period, with users submitting multiple images over this period (average = ~9.3 (SD = 11.4) images per user [23]). Of these, 745 were excluded due to being, practice images taken during onboarding, an older one-strip test version identified as unused, and duplicate submissions caused by early app issues that allowed duplicate uploads when users were uncertain whether their image had been sent. This left 1,655 images for analysis.

Among the analyzed images (Table 2), 833 (50%) were determined to be **positive** for isoniazid metabolites (indicated by a color change in both test and control lines), 366 (22%) were classified as **negative**) (showing a color change for control line only, with no corresponding change in the test line), and 449 (27%) were **failed/inconclusive** tests (characterized by no color change in the control line (white or urine-colored—yellow/orange)). Seven (0.4%) were deemed **uninterpretable** due to issues such as test line not being captured or cartridge flooding. Test images classified as negative or inconclusive required that the treatment supporter follow up with the participant to request a follow up test or provide support regarding medication adherence. There was high agreement between the two raters; the overall Kappa was 0.89 [0.78 – 0.98] (Cohen’s unweighted kappa with 1,000-bootstrap 95% confidence intervals) (S1 Table).

**Table 2:** Count of the clinical interpretation of test results.

Result	Color of Test and Control Line	Interpretation	Number of Tests (N = 1,655)	Percentage of Total (%)
Positive	Test line and control line are blue/purple	Metabolite was detected. Interpreted as medication was taken within the last 24 hours.	833	50%
Negative	Test line is White/No color/yellow and control line is blue purple	Metabolite not detected in the sample. Interpretation that medication was likely not taken within the last 24 hours. Need to follow up with participant.	366	22%
Failed/Inconclusive	Control line without color change of blue/purple	Unable to determine result (possibly did not perform test as instructed or test issue)	449	27%
Uninterpretable	Test window not fully visible for test and control line	Unable to categorize (test window wasn’t clearly visible in photo)	7	0.4%

Even when images could be determined to be positive, negative, or inconclusive, they could also have one or more identified issues (e.g., positive and poor lighting). Across all analyzable images (Table 3): 424 test lines (26%) and 317 (19%) control lines showed color variations (e.g., colors other than blue-purple), 684 images (41%) had photo quality issues (e.g., poor lighting, blurriness, distance), and 329 images (20%) had test procedure or cartridge-related issues (e.g., flooding, sticker damage, cartridge separation). Issues could co-occur, with the most overlaps seen with flooding of the test cartridge and another issue. The sections below provide a more detailed analysis of these issues, organized by: (1) color variation, (2) photo quality, and (3) mechanical/cartridge-related problems.

**Table 3:** Count of identified issues.

Issue	Number of Tests (N = 1,655)	Percentage of Total
Positive Test Line Color Variation	424	26%
Control Line Variation	317	19%
Photo Quality	684	41%
Test Procedure/Cartridge Related	329	20%

### 3.3. Color variations in test and control lines

The expected positive result was a color change to blue-purple in both control and test lines. Among **control line** color variations not blue/purple or white (Table 4), 165 images (10%) were either pink/magenta, green/blue, or a color gradient. A total of 152 images (9%) showed control lines with urine-colored hues (yellow/orange/red) (Fig 2A-D).

**Table 4:** Count of color variations.

Line & Color Variation	Number of Tests (N = 1,655)	Percentage of Total
Control Line- Pink/Magenta, Green/Blue, Gradient	165	10%
Control Line- Urine Colored	152	9%
Test Line- Pink/Magenta, Green/Teal, Gradient	54	3%
Test Line- Color Change only at Top	196	12%
Test Line- Urine Colored	174	11%

**Fig 2:**
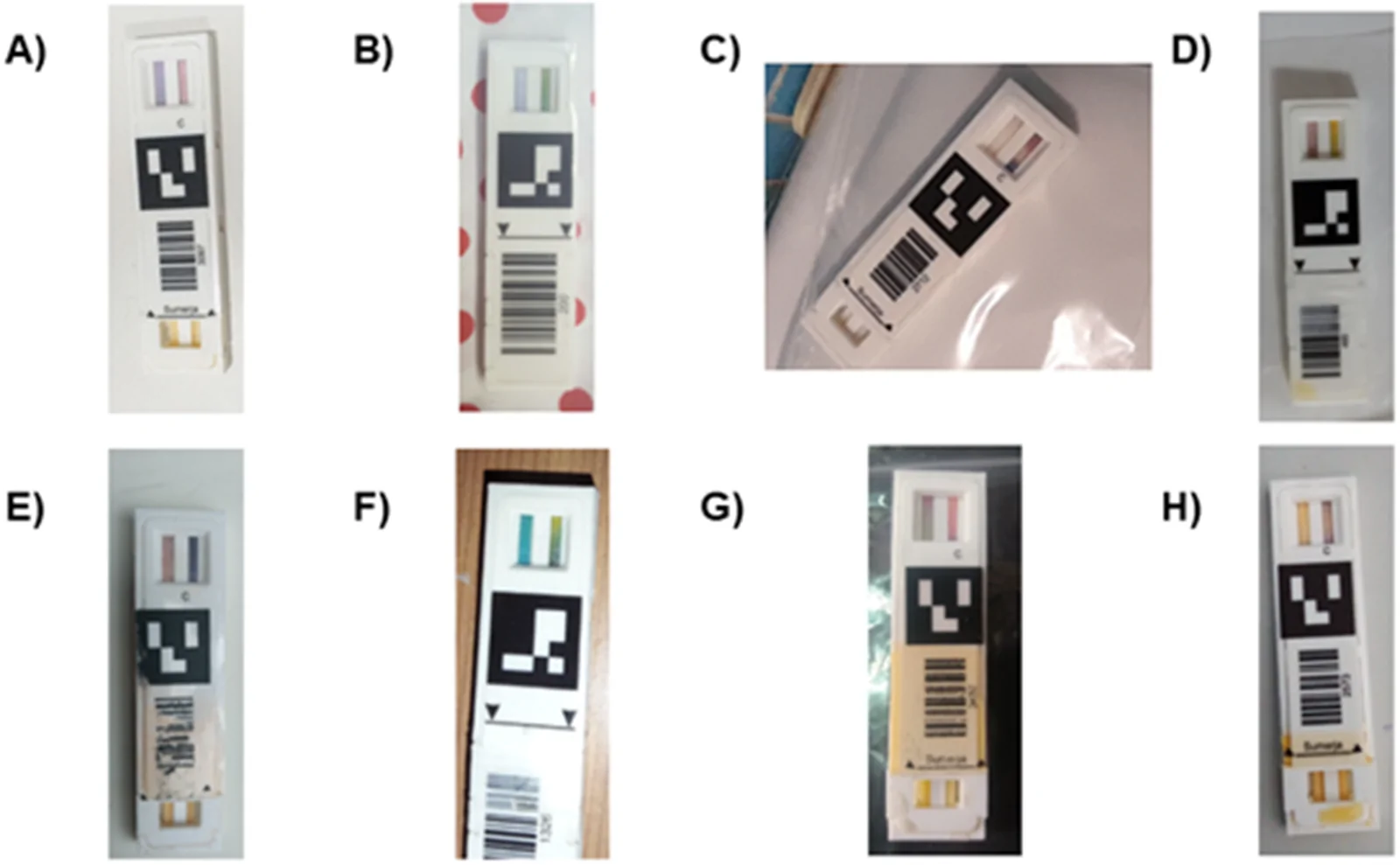
Representative images of color variations. Control line: A: Pink; B: Green; C: Gradient; D: Yellow. Test line: E: Pink; F: Green; G: Possible Contamination; H: Yellow.

For **test line** color variations not blue/purple or white (Table 4), 54 (3%) were pink/magenta, green/teal hues, and gradient effects. 196 (12%) displayed a thin line of color change only at the top of the test strip. 174 (11%) images showed urine-colored hues (Fig 2E-H).

### 3.4. Photo quality issues

Of the 684 images with photo quality issues (Table 5), the most common problems, ranked in order of frequency, were: blurry image – lack of sharpness reducing result clarity (234, 34%); incomplete test capture – only part of the test was visible in the image (176, 26%); poor lighting – shadows, too bright (i.e., flash), or low light obscured test results (134, 20%); distant shots – the test was photographed from too far away (110, 16%); and test inside storage bag – the plastic covering cause glare or distortion, making interpretation more difficult (30, 4%) (Fig 3).

**Table 5:** Count of photo quality issues.

Issue	Number with Issue (n = 684)	Percentage of Total
Blurry- Lack of Sharpness	234	34%
Incomplete Capture of Test	176	26%
Poor Lighting	134	20%
Distant Shots	110	16%
Distortion caused by plastic storage bag	30	4%

**Fig 3:**
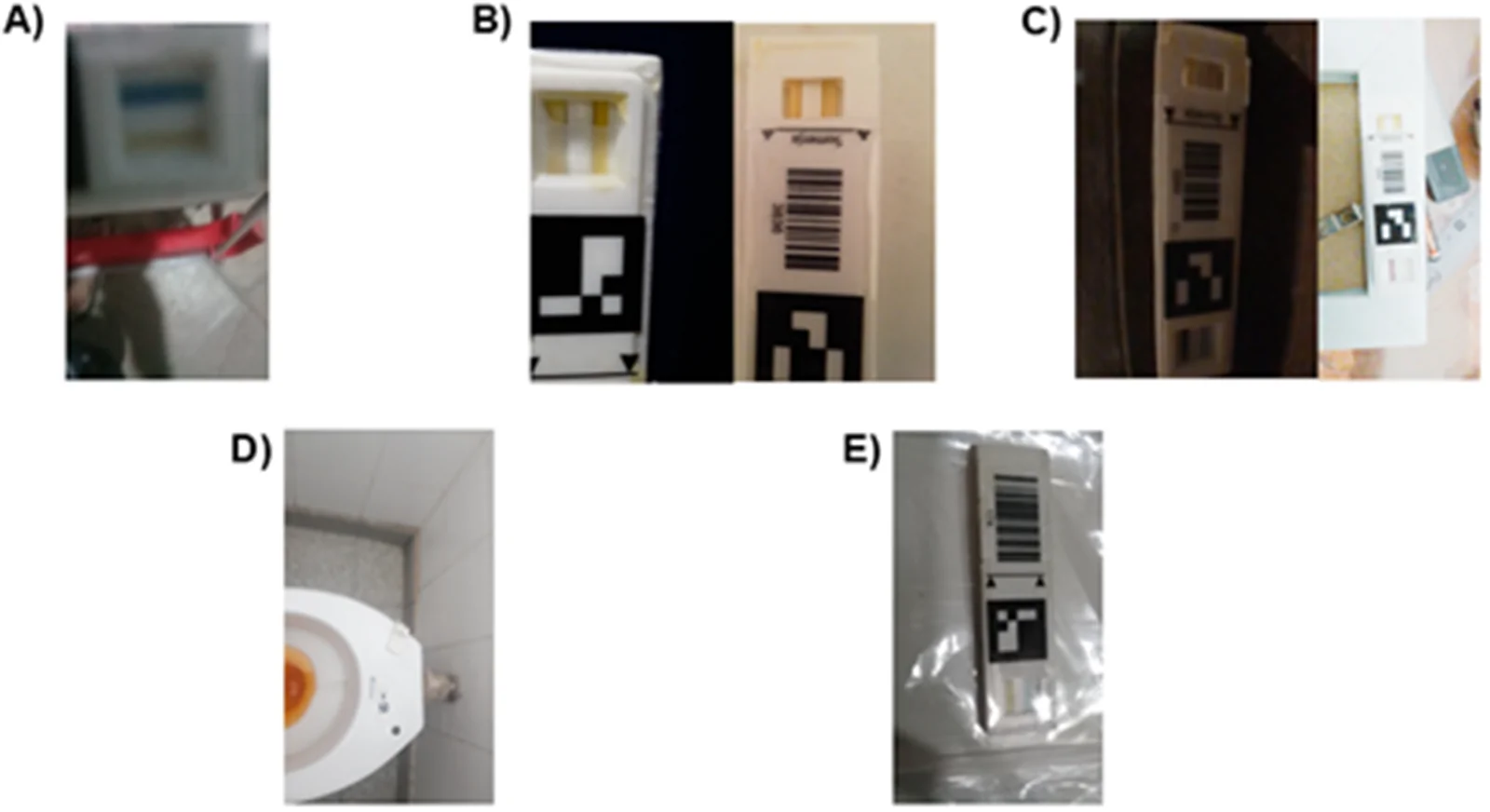
Representative images of photo quality issues. A: blurry image and incomplete test capture; B: incomplete test capture; C: poor lighting; D: distant shot; E: test inside storage bag.

### 3.5 Cartridge or test procedure issues

Of the 329 images with cartridge-related issues that affected test interpretation (Table 6), the four most common problems included: possible contamination – color change at only the top of the test line (196, 12%); flooding – the void window was oversaturated with urine, likely interfering with the reaction (83, 5%); sticker damaged – the cartridge sticker was compromised or detached (48, 3%); and cartridge separation – hardware components became loose or detached (2, 0.1%) (Fig 4). A possible cause for a color change only at the top of the test line (rather than the full test line) was determined to be fluid wicking from control line.

**Table 6:** Cartridge or test procedure issues.

Issue	Number with Issue (n = 329)	Percentage with Issue
Color Change at Top of Test Line	196	60%
Flooding	83	25%
Sticker Damaged	48	15%
Hardware Detached	2	1%

**Fig 4:**
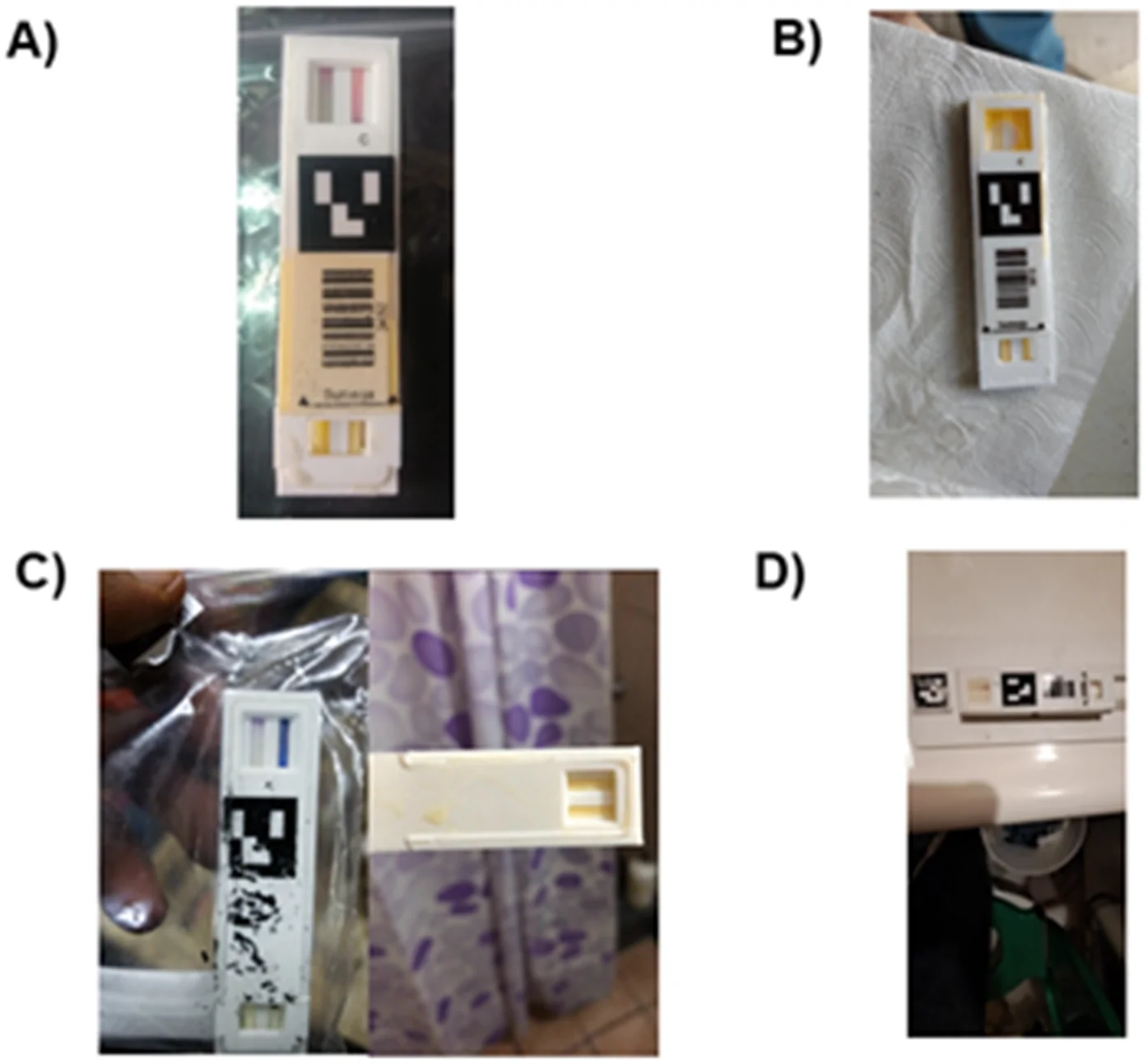
Representative images of test cartridge issues. A: Possible contamination; B: Flooded; C: Sticker damaged; D: Cartridge separation.

## 4. Discussion

This study evaluated user-submitted images of the TB-TST LFA used in a pragmatic randomized controlled trial [12]. Despite some images having multiple issues, only 0.4% were unreadable supporting feasibility and usability of the home-based test results being uploaded through an app. Previous studies have analyzed user-submitted photos for other health conditions [24–26] and some have explored the challenges users face in capturing and uploading these images [27]. Understanding these challenges is critical for designing user-friendly technologies and improving intervention efficacy. While quantitative analyses assess intervention success, qualitative exploration of color interpretability offers complementary insights.

Existing social science and user-centered design frameworks primarily focus on mobile application usability [28]. In this study, we integrated social sciences methodologies and engineering design principles to improve the LFA’s interpretability. Image analysis methodologies remain underutilized in health research [29]; but offer valuable insights into how users interpret tests and follow instructions. Integrating image-based qualitative analysis can inform the development of more user-centered technologies.

### 4.1. Color variation and contributing factors

Most test results were clear positives, showing a blue or purple color change, confirming the presence of isoniazid metabolites in the urine. However, inconclusive results required follow-up from a medical professional, highlighting the importance of understanding issues and color variations. The most common unclear result was white-colored test strip, which could indicate an unused test (i.e., no exposure to urine), potential non-adherence (i.e., participant did not take medication in past 24 hours), improper test timing (e.g., taking the picture too soon), incorrect test procedure (e.g., test flipped on end/upside down/not run on a flat surface), or environmental factors (e.g., humidity, light exposure affecting reactivity). Since the test is designed to detect recent medication ingestion (within 24 hours), deviations from this timing may lead to false-negative results. Procedure errors such as incorrect test orientation (e.g., tests not run on a flat surface) could have hindered the ability of the chemical reaction to occur. Environmental factors such as humidity can also reduce test reactivity, emphasizing the need for moisture barrier packaging and desiccants to maintain test integrity. Although desiccants were provided with the tests, it is possible that they could have been removed or needed more if stored in more humid environments such as bathrooms. Addressing these factors through improved manufacturing, patient education, and clearer instructions could reduce the occurrence of unclear results.

Color deviations, such as teal, red, pink, or yellow, were observed. Prior studies suggest color variations may result from external factors, including nicotine metabolites, which may undergo similar chemical reactions as isoniazid, producing blue-green hues [8,30]. Rifampicin, commonly co-administered for TB treatment, turns urine dark orange [31]. In addition, time-dependent color shifts have been observed, with progressive changes from purple to blue, green and yellow over 72 hours [30,32]. Subbaraman, et al. found that a yellow result indicated that the medication was not taken within the prior 48 – 72 hours [33].

Another potential factor influencing color variation is the pH-dependent halochromism of cyanine dyes, the reaction product in the test [8,34]. Cyanine dyes are sensitive to pH changes, leading to shifts in color intensity and hue [35]. Although we did not collect dose timing or urine pH data in this study, these findings suggest the need for better patient education on test timing, clearer guidelines for interpreting colorimetric results, and further studies on the impact of nicotine metabolites, pH-dependent color changes, time-dependent chemistry, and the role of buffering agents in test chemistry.

### 4.2. Photo quality issues and solutions

Approximately 40% of user-submitted photos were difficult to evaluate due to blurriness (e.g., camera movement, improper focus), incomplete test capture (e.g., partial test visibility), poor lighting (e.g., shadows, overexposure), distant shots (e.g., taken too far from the test), and test photographed inside the storage bag, causing glare or distortion. Although photographing the test inside the plastic bag did not always affect visibility, these findings emphasize the need for better user guidance and education to optimize test use. Similar image quality challenges have been reported in other mobile health applications, particularly among users with limited phone experience or physical impairments [22]. To improve test quality, future iterations of the mobile application could incorporate: automated image pre-screening to detect low-quality images before submission [36], reference charts for color calibration and lighting standardization [37], and visual guides for proper image positioning and framing [38].

### 4.3. Cartridge quality issues

Several test cartridge issues were identified, including potential contamination or color wicking at the top of the test line, flooding, sticker damage, and cartridge separation from structural failure of test components. These issues resulted from potentially both user errors (e.g., flooding) and manufacturing defects (e.g., cartridge separation). Contamination, where color from the control line potentially migrated to the top of the test line likely resulted from capillary action along the test cartridge. To mitigate these issues, we propose the following design improvements: an additional sample pad to regulate sample volume, prevent flooding, and control sample flow [39], directly printing information on the cartridge instead of using stickers to minimize damage, and refining the assembly process to improve cartridge durability and prevent structural failures.

## 5. Limitations

Several limitations should be noted. First, images were separately and did not include the self-reported data on time of medication intake limiting our ability to thoroughly analyze the reasons for the color variations observed. Second, qualitative analysis of the images may be more subjective versus an automated color detection, although this was representative of real-world conditions with this iteration of the TB-TST LFT in which health workers analyze the results manually. Based on study findings, we aim to optimize test performance and usability, better support patient treatment adherence, and improve decision-making for allocation of scarce healthcare resources. By integrating user-centered design principles with engineering innovation, we can improve accessibility, reliability, and real-world impact of such technologies in global health settings.

## 6. Conclusions

This study evaluated the successes and challenges of real-world use of a colorimetric TB treatment adherence assay. Given the subjective nature of colorimetric-based tests, optimizing both user experience and evaluator reliability is crucial. From our analysis of user submission issues as well as test quality issues, we identified key areas for improvement include user guidance (e.g., improved educational materials, visual aids), test cartridge design (e.g., contamination prevention, enhance manufacturing protocols), interpretation protocols (e.g., clearer criteria for intermediate color changes) and automated test reading. Although there were some challenges to submitting a clear image of the test, few were unreadable, indicating the overall success of user-submitted test images. The TB-TST LFA represents an innovative approach to supporting TB treatment adherence, with potential for significant global health implications. Insights from this study inform future test optimization and provide valuable guidance for the development of other point-of-care tests using colorimetric detection.

## Supporting information

S1 File Contains the codebook utilized by the researchers to classify the user-uploaded images. (DOCX)

S1 Table Inter-rater agreement for TB test strip classification (N = 77). (DOCX)
