# Supplementary material for: Evaluating a biochemical TB medication adherence test: Insights from image-based reporting through a patient companion app
Source: PLOS Glob Public Health. 2026 Oct 5;6(10):e0007182. doi: 10.1371/journal.pgph.0007182 (PMC13637889; doi:10.1371/journal.pgph.0007182)
Supplement: Supplementary file 1 [file pgph.0007182.s001.docx]

Supplemental Document 1: Codebook for Image Analysis

Definitions:

- Control line: Line on the right when the test is oriented such that the void window is at the bottom and the test window is at the top.
- Test line: Line on the left when the test is oriented such that the void window is at the bottom and the test window is at the top.

Codebook:

| Group | Code | Description | Example Image |
| --- | --- | --- | --- |
| Test & Control Line | Unused | Test shows still being placed inside bag or with no signs of usage with white test and control line. |  |
|  | No Issue Test/Control Line | Dark blue control line with a lighter blue test line. |  |
| Test Line | Yellow/Orange/Red Test Line | Test line appears to be a yellow/orange/red color. |  |
|  | Pink/Purple ONLY at Top of Test Line | Pink/Purple at top of test line shows to be caused by contamination while the remainder of test line appear different color. |  |
|  | White Test Line | Test line appears white while control line did have a change/reaction. |  |
|  | Normal Test Line | Test line shows to be a darker blue color, which means normal while control line appears not normal. |  |
|  | Other Test Line | Test line does not fit any other categorization and with random color changes. |  |
| Control Line | Blue/Purple/Light Color Control Line | Control line shows to be a blue/purple/lighter which means normal while test line appears not normal. |  |
|  | White or No Color Change Control Line | Control line appears white or a yellow color from signs of being used but with no change in control line. |  |
|  | Yellow Control Line | Control line appears to be a yellow color. |  |
|  | Other Control Line | Control line does not fit any other categorization and with random color changes. |  |
| Photo Quality Issues | Blurry | Image is not clearly visible due to a lack of clarity and sharpness. |  |
|  | Far Away/Bad Angle | Image is taken from too far and features of the test are not able to be read, understood, or seen. |  |
|  | Issue- Photographed in Bag | Image shows the test still placed inside of bag which it is delivered in. The bag may cause an issue to clearly read and understand the test. |  |
|  | Flash Reflection | Image was taken with the photographer’s flash on. The flash caused an imbalance in lighting and made it difficult to properly read test results. |  |
|  | Lighting/Shadow/Glare | Image was taken in inappropriate lighting making it difficult to accurately read test results. |  |
| Test Quality Issues | Flooded | Test was not done accurately or correctly, which leaves a flooding of urine in the void or test window. |  |
|  | Sticker Damaged | Test sticker is showing to be notably damaged in the image. |  |
|  | Cartridge Separation | Test cartridge is separating and/or breaking apart. |  |
| Other | Results not in Image | Image was taken in a form which excludes the control and test line from the full image or other features are missing from image like a barcode which makes it unable to be read. |  |
|  | Not Full Test in Image | Image was taken in a form that excludes parts of the full test making the results unable to be processed. |  |
|  | Excluded Images | Images excluded from revision process because test was not included in image. |  |
