# Supplementary material for: Evaluating a biochemical TB medication adherence test: Insights from image-based reporting through a patient companion app
Source: PLOS Glob Public Health. 2026 Oct 5;6(10):e0007182. doi: 10.1371/journal.pgph.0007182 (PMC13637889; doi:10.1371/journal.pgph.0007182)
Supplement: Supplementary file 2 [file pgph.0007182.s002.docx]

S1 Table. Inter-rater agreement for TB test strip classification (N = 77)

Cohen's unweighted kappa with 1,000-bootstrap 95% confidence intervals

| Category | Code | Observed agreement | Expected agreement | κ (95% CI) | Interpretation |
| --- | --- | --- | --- | --- | --- |
| Overall | — | 93.51% | 37.88% | 0.896 (0.786, 0.983)^a^ | Almost perfect |
|  | | | | | |
| Positive | 1 | 98.70% | 56.60% | 0.970 | Almost perfect |
| Negative | 2 | 98.70% | 82.41% | 0.926 | Almost perfect |
| Failed / Inconclusive | 3 | 93.51% | 50.03% | 0.870 | Almost perfect |
| Uninterpretable | 4 | 96.10% | 86.73% | 0.706 | Substantial |

^a^ Bias-corrected (BC) bootstrap 95% CI from 1,000 replications

Category-specific κ values are derived from binary recoding (category present vs. absent); 95% CIs not bootstrapped individually.
Kappa interpreted using Landis & Koch (1977): <0.20 slight; 0.21–0.40 fair; 0.41–0.60 moderate; 0.61–0.80 substantial; >0.80 almost perfect
